# Melatonin ameliorates *Slc26a2*-associated chondrodysplasias by attenuating endoplasmic reticulum stress and apoptosis of chondrocytes

**DOI:** 10.1016/j.gendis.2024.101350

**Published:** 2024-06-14

**Authors:** Pan Li, Chao Zheng, Jingyan Hu, Weiguang Lu, Dong Wang, Xue Hao, Chengxiang Zhao, Liu Yang, Zhuojing Luo, Qiang Jie

**Affiliations:** a Institute of Orthopedic Surgery, Xijing Hospital, Fourth Military Medical University, Xi'an, Shaanxi 710032, China; b Pediatric Orthopaedic Hospital, Honghui Hospital, Xi'an Jiaotong University, Xi'an, Shaanxi 710032, China; c Research Center for Skeletal Developmental Deformity and Injury Repair, College of Life Sciences and Medicine, Northwestern University, Xi'an, Shaanxi 710032, China; d Xi'an Key Laboratory of Skeletal Development Deformity and Injury Repair, Xi'an, Shaanxi 710032, China

**Keywords:** Ca^2+^overload, Chondrodysplasia, Endoplasmic reticulum stress, Melatonin, SLC26A2

## Abstract

Although the pathogenesis and mechanism of congenital skeletal dysplasia are better understood, progress in drug development and intervention research remains limited. Here we report that melatonin treatment elicits a mitigating effect on skeletal abnormalities caused by *SLC26A2* deficiency. In addition to our previous finding of endoplasmic reticulum stress upon *SLC26A2* deficiency, we found calcium (Ca^2+^) overload jointly contributed to *SLC26A2*-associated chondrodysplasias. Continuous endoplasmic reticulum stress and cytosolic Ca^2+^ overload in turn triggered apoptosis of growth plate chondrocytes. Melatonin, known for its anti-oxidant and anti-inflammatory properties, emerged as a promising therapeutic approach in our study, which enhanced survival, proliferation, and maturation of chondrocytes by attenuating endoplasmic reticulum stress and Ca^2+^ overload. Our findings not only demonstrated the efficacy of melatonin in ameliorating abnormal function and cell fate of *SLC26A2*-deficient chondrocytes *in vitro* but also underscored its role in partially alleviating the skeletal dysplasia seen in *Col2a1-CreER*^*T2*^; *Slc26a2*^*fl/fl*^ mice. As revealed by histology and micro-CT analyses, melatonin significantly improved retarded cartilage growth, defective trabecular bone formation, and tibial genu varum *in vivo*. Collectively, these data shed translational insights for drug development and support melatonin as a potential treatment for *SLC26A2*-related chondrodysplasias.

## Introduction

Solute carrier family 26 member 2 (*SLC26A2*) encodes a cell membrane protein that functions as a SO_4_^2−^ transporter, facilitating the uptake of inorganic sulfate into cells.^1^ Mutations in *SLC26A2* lead to chondrodysplasia, a rare genetic disorder characterized by abnormal cartilage development that affects bone growth.^2^^,^^3^ Individuals with *SLC26A2* mutations may exhibit various skeletal abnormalities, including short stature, limb deformities, joint problems, and distinctive facial appearance.^4^ The severity of symptoms can vary widely, leading to a spectrum of skeletal dysplasia disorders, from lethal achondrogenesis type 1B to relatively milder diastrophic dysplasia.^5^ Unfortunately, the current challenge lies in the absence of effective pharmaceutical interventions for *SLC26A2*-associated chondrodysplasias.

In our previous study, we generated *Slc26a2* knockout mice using vasa-Cre and *Slc26a2* floxed mice and found obvious endoplasmic reticulum (ER) stress in growth plate chondrocytes with abundant collagen retention in the ER lumen.^2^ ER stress then triggered the unfolded protein response (UPR), where the transcription factor 6 (ATF6) arm of the UPR was more profoundly activated upon *SLC26A2* deficiency. Following this, the N-terminus of ATF6 translocated into the cell nucleus and transactivated fibroblast growth factor receptor 3 (FGFR3), a receptor mediating inhibitory regulation of cartilage growth.^6^ Although subsequent intervention with FGFR3 inhibitors partially rescued the cartilage phenotype of *Slc26a2* total knockout mice, the mutant pups still showed abnormal skeletal growth and survived only a limited time after birth,^2^ which led us to the speculation that other responsive machinery downstream ER stress might jointly contribute to the pathogenesis of *SLC26A2* deficiency in chondrocytes. Nevertheless, based on the hallmarks of ER stress seen in growth plate chondrocytes of *Slc26a2* knockout mice, drugs that broadly target and alleviate ER stress might improve the skeletal defects.

Melatonin, an indoleamine hormone primarily secreted by the mammalian pineal gland, has been linked to the control of diverse pathophysiological processes, such as circadian rhythm, tumor growth inhibition, immune response modulation, apoptosis, and autophagy.^7^, ^8^, ^9^ Although melatonin is known for its association with circadian rhythms and sleep, recent research has highlighted its protective effects on cell survival against ER stress.^10^ Several studies have indicated that melatonin may positively influence ER function by alleviating ER stress; down-regulating the expression of ER stress signatures, such as phosphorylated inositol-requiring enzyme type 1 (p-IRE1), phosphorylated protein kinase RNA-like ER kinase (p-PERK), and activating transcription factor 4 (ATF4).^11^^,^^12^ Specifically, melatonin effectively mitigates ER stress-induced pro-apoptotic effects in bone mesenchymal stem cells during mitochondrial oxidative damage by activating the AMP-activated protein kinase (AMPK) pathway.^13^ Additionally, it up-regulates sirtuin 1 (SIRT1) expression and dampens IRE1α–XBP1S (the spliced form of X-box binding protein 1)–CHOP (C/EBP homologous protein) activity to alleviate ER stress-induced apoptosis in chondrocytes.^14^ Moreover, melatonin boosts the survival and function of transplanted senescent canine stem cells by activating Nrf2 and inhibiting ER stress, underscoring its multifaceted protective role against cellular stress.^15^ Given that, we aimed to investigate the therapeutic effects of melatonin on *SLC26A2*-deficient chondrodysplasias in the present study.

This study delved into the mitigating effects of melatonin on defective growth of cartilage and bone caused by *SLC26A2* deficiency, with a specific emphasis on its effects on non-lethal chondrodysplasias via postnatal administration. By testing on *Slc26a2*-deficient chondrocytes *in vitro* and *Col2a1-CreER*^*T2*^;*Slc26a2*^*fl/fl*^ mice *in vivo*, we observed that melatonin alleviated ER stress and abnormal function and cell fate of chondrocytes upon *SLC26A2* deficiency, leading to an evident improvement of retarded cartilage growth, defective trabecular bone formation, and tibial genu varum. Besides, our findings indicated that ER stress led to an increased concentration of intracellular free Ca^2+^, indicative of Ca^2+^ overload, jointly contributing to chondrocyte apoptosis. Moreover, melatonin effectively restored the Ca^2+^ concentration balance in *Slc26a2*-deficient chondrocytes. These data support melatonin as a potential treatment to ameliorate *SLC26A2*-related chondrodysplasias.

## Materials and methods

### Animal models

*Slc26a2*^*fl/fl*^ mice were constructed by Cyagen (Suzhou) Biotechnology Co. (order number TOS151108AJ2). Professor Di Chen generously gifted col2a1-CreERT2 mouse strain from the Shenzhen Institutes of Advanced Technology.^16^ To reduce the likelihood of genetic background influence, the mouse strains utilized in this investigation underwent nine generations of crossing with wild-type C57BL/6 mice before any assessments were initiated. Animal care and experimentation were carried out according to protocols approved by the Animal Research Ethics Committee of the Fourth Military Medical University.

### Safranin O staining

To perform Safranin O staining, tibial tissues underwent fixation in 4% paraformaldehyde overnight. Subsequently, the tissues were embedded in an optimal cutting temperature compound (Leica), cryosectioned at 8 μm, and stained with safranin O and fast green.

### Chondrocyte culture

To acquire primary chondrocytes, rib cartilage was obtained from E18.5 (embryonic day 18.5) embryos and digested with collagenase D (Roche) at a concentration of 3 mg/mL. The digestion process lasted for 40 min at 37 °C in a thermal incubator with 5% CO_2_. Tissue debris was stirred several times to detach the soft tissue and then transferred to 0.5 mg/mL collagenase D solution at 37 °C overnight. Collection of the digest was done through a 40 μm cell filter and centrifuged at 500 *g* for 10 min. The resulting precipitate was resuspended in Dulbecco's modified Eagle medium (Sigma–Aldrich) supplemented with 10% fetal bovine serum, 2 μM L-GIn, 50 U/mL penicillin, and 0.05 mg/mL streptomycin. The primary chondrocytes were cultured in a sterile environment at 37 °C and 5% CO_2_, and seeded at a density of 25 × 10^3^ cells per square centimeter.

### CCK-8 experiment

Cell proliferation was analyzed through the utilization of the Cell Counting Kit-8 (CCK8) obtained from Beyotime (Shanghai, China). Chondrocytes were initially placed in 96-well dishes with a concentration of 5000 cells per well and incubated in a 37 °C incubator with 5% CO_2_ to allow adherence to the surface. Following a 72-h incubation, each well received 10 μL of CCK-8 solution and underwent further incubation at 37 °C with 5% CO_2_ for 1 h. Subsequently, the optical density of viable cells was measured at 450 nm using microplate readers to evaluate each sample's absorbance.

### Apoptosis assay

For TUNEL assays, cultures and tibial sections were subjected to TUNEL staining, utilizing a Beyotime Biotechnology kit, following the guidelines provided by the manufacturer. Apoptosis of cultured chondrocytes was also examined by utilizing a flow cytometer with an Annexin V-FITC/PI Apoptosis Detection Kit (BD Biosciences, USA). Processed cells were harvested with 0.25% trypsin, subjected to three washes with cold phosphate buffer saline solution, and then suspended in staining buffer. To evaluate the apoptosis of chondrocytes, flow cytometry was conducted after annexin V-FITC/PI staining.

### Immunostaining

Immunofluorescence analysis of the frozen tibial sections was conducted according to previously established procedures.^2^ Ki67 antibody (Millipore, Burlington, MA, USA, 1:100), anti-COL X (Abclonal, A11645, 1:100), anti-SOX9 (SRY-box transcription factor 9) (Abcam, ab185966, 1:100), anti-COL II (collagen type II) (Thermo Fisher, PA1-26206, 1:100), and anti-Aggrecan (Thermo Fisher, MA3-16888, 1:100) were the primary antibodies utilized. To extract Col X (collagen X) antigens, slices were digested with 2 mg/mL dilution of hyaluronidase (Sigma Aldrich) at 37 °C for 20 min. The suitable Alexa Fluor-conjugated secondary antibody (Abcam) was utilized to detect the primary antibodies. The ProLong Gold Antifade was used to visualize all sections, which were then examined under a fluorescence microscope (Zeiss).

### Flow cytometry analysis and Fluo-3 AM fluorescence staining

We employed Fluo-3 AM, a fluorescent Ca^2+^ indicator, to assess intracellular calcium concentration, and BAPTA-AM, a cell-permeable Ca^2+^ chelator, to establish a negative control by chelating intracellular Ca^2+^. To prepare the Fluo-3 AM (Invitrogen Co., USA) stock solution (5 mM), the Pluronic F-127 at 20% (w/v) in absolute dimethyl sulfoxide was used. To achieve a working concentration of 5 μM, Hank's balanced salt solution was used to dilute an isotope of the specimen containing 0.39 g/L KCl, 0.07 g/L KH_2_PO_4_, 8.06 g/L NaCl, 0.10 g/L Na_2_HPO_4_·7H_2_O, 0.24 g/L CaCl_2_, 0.10 g/L MgCl_2_, 0.10 g/L MgSO_4_, and 1.52 g/L d-glucose. This dilution step ensures that the cells are maintained in an isotonic environment, optimal for preserving cell viability. For accurate intracellular Ca^2+^ level measurement via flow cytometry, the forward scatter gain was adjusted to E-00 and the side scatter to 262 on the FACS Calibur. The FL1 fluorescence detection channel was set to logarithmic mode to enhance the detection of the Fluo-3 AM signal. Approximately 2 × 10^6^ cells were then suspended in 300 μL of Hank's balanced salt solution and placed into a FACS sample tube for analysis at an excitation wavelength of 488 nm and emission wavelength of 520 nm.^17^ The Fluo-3 AM fluorescence staining, aimed at quantifying Ca^2+^ concentrations, encompasses two crucial steps, loading and de-esterification. Cells were incubated with 5 μM Fluo-3 AM at varied temperatures (4 °C, 20 °C, and 37 °C) to ensure optimal dye uptake. Post-loading, cells were thoroughly washed with undiluted Hank's balanced salt solution and incubated at 37 °C for 30 min to complete the de-esterification process, activating the dye for Ca^2+^ binding. Following this procedure, cells were examined under a fluorescence microscope (Zeiss), allowing for the detailed observation of intracellular Ca^2+^ dynamics.^18^

### Endoplasmic reticulum fluorescence staining labeling

ER-Tracker Red (Beyotime) was added to the dilution solution at a ratio of 1:1000 and mixed thoroughly to obtain the ER-Tracker Red working solution. Afterward, each group of cells was incubated at 37 °C for 30 min with this working solution. Following incubation, the working solution of ER-Tracker Red staining was removed from cells before they were photographed.

### Western blot and quantitative real-time reverse transcription PCR

For the immunoblotting assays, tissues and cells were lysed by RIPA buffer and centrifuged to extract total proteins. Protein concentration was determined using the Pierce BCA Protein Assay Kit (Thermo Fisher Scientific). A certain amount of protein was mixed with loading buffer (Beyotime Biotechnology), boiled for 15 min, and subjected to SDS-PAGE followed by transferring to PVDF membranes. Blots were probed with primary antibodies, including glyceraldehyde 3-phosphate dehydrogenase (GAPDH) (Cell Signaling Technology, #2118, 1:2000), anti-SLC26A2 (Abclonal, #A6369, 1:1000), anti-ATF6 (Abcam, #ab37149 1:1000), anti-BIP (Cell Signaling Technology, #3183, 1:1000), anti-ATF4 (Cell Signaling Technology, #11815, 1:1000), anti-XBP1(Abcam, #ab37152, 1:1000), anti-CHOP (Cell Signaling Technology, #2895, 1:1000) CASPASE 3 (Cell Signaling Technology, #9662, 1:1000), and cleaved CASPASE-3 (Cell Signaling Technology, # 9661, 1:1000). To perform quantitative PCR, the MiniBEST Universal RNA Extraction Kit (TaKaRa) was utilized for extracting total RNA from tissues or cells. Reverse transcription was performed with PrimeScript RT Master Mix (TaKaRa). Synthesized cDNA was subjected to quantitative PCR analysis using TB Green Premix Ex Taq II (TaKaRa). The primers used are listed in Table 1.

**Table 1:** Primers used for qPCR.

Gene	Primer sequences (5′-3′)
*Gapdh* (F)	AGCTACTCGCGGCTTTACG
*Gapdh* (R)	ATCCGTTCACACCGACCTTC
*Slc26a2* (F)	AAGAGCAGCATGACCTCTCAC
*Slc26a2* (R)	CTGCCTCAAGTCAGTGCCT
*Sox9* (F)	GAGTTTGACCAATACTTGCCAC
*Sox9* (R)	ACTGCCAGTGTAGGTGAC
*Col2a1* (F)	ATCTTGCCGCATCTGTGTGT
*Col2a1* (R)	CTCCTTTCTGCCCCTTTGGC
*Aggrecan* (F)	GCCTACCCGGTACCCTACAG
*Aggrecan* (R)	ACATTGCTCCTGGTCTGCAA
*Xbp1* (F)	AAGAGGACCTGTGGCTTGTG
*Xbp1* (R)	TGTGACTGTAGTACACATGTCTGG
*Atf4* (F)	CAACCTATAAAGGCTTGCGGC
*Atf4* (R)	CCAACACTTCGCTGTTCAGG
*Atf6* (F)	AAGAGGACCTGTGGCTTGTG
*Atf6* (R)	TGTGACTGTAGTACACATGTCTGG
*Bip* (F)	GTGTGTGAGACCAGAACCGT
*Bip* (R)	GTTCTTGAACACACCGACGC

### Drug treatment

Primary chondrocytes isolated from *Col2a1-CreER*^*T2*^;*Slc26a2*^*fl/fl*^ mice were treated for 48 h with 1 μM 4-hydroxy tamoxifen, an active metabolite of tamoxifen. Melatonin, dissolved in dimethyl sulfoxide, was applied at different concentrations as indicated in the figures. For the induction of Cre recombination in mice, tamoxifen (Sigma–Aldrich, St. Louis, MO, USA) was dissolved in corn oil at a concentration of 10 mg/mL. To eliminate *Slc26a2* expression in chondrocytes, tamoxifen (1 mg per 10 g body weight) was intraperitoneally administered to *Col2a1-CreER*^*T2*^;*Slc26a2*^*fl/fl*^ (*Slc26a2* cKO) mice and wild-type (cre-negative control) littermates. Melatonin was daily administered at a dosage of 2 mg/kg of body weight, starting from P20 (postnatal day 20) to P49, except for the day when tamoxifen was administered.

### Micro-CT analysis

Faxitron machine (MX-20) was used for radiographing mouse tibias. Following the elimination of soft tissues, entire mouse skeletal structures, including tibias and femurs, were acquired. Then samples were fixed in 4% paraformaldehyde at 4 °C for one day and subsequently stored in 70% ethanol. The samples were placed inside a tailored scanning tube with the help of a foam plate. The scanning parameters were configured as follows (eXplore Locus SP; GE Healthcare Co., USA): scanning resolution (21 μm), rotation angle (360°), rotation angle increment (0.4°), voltage (80 kV), current (80 μA), exposure time (3000 ms), frame average (4), and pixel combination (1 × 1). The calibrations for scanning in black and white involved adjusting the Hounsfield scale and correcting streak normalization. Subsequently, the scanned documents underwent 3D reconstruction using Micview V2.1.2 software for processing and ABA software for further analyses.

### Statistical analysis

GraphPad Prism 8.0 (GraphPad Software, USA) was employed for statistical analyses. The results were expressed as mean ± standard deviation. An F-test was conducted to test for the equality of variances. The normal distribution of the collected data was assessed using the Shapiro–Wilk test or D'Agostino test. After the analysis with one-way analysis of variance (ANOVA), Tukey's post-hoc test was employed to compare means among different groups. The variable *n* represents the number of replicates or mice assigned to each group. The threshold for determining statistical significance was set at *P* < 0.05.

## Results

### Melatonin ameliorates multiple chondrocyte defects caused by *Slc26a2* deficiency

**Figure 1:**
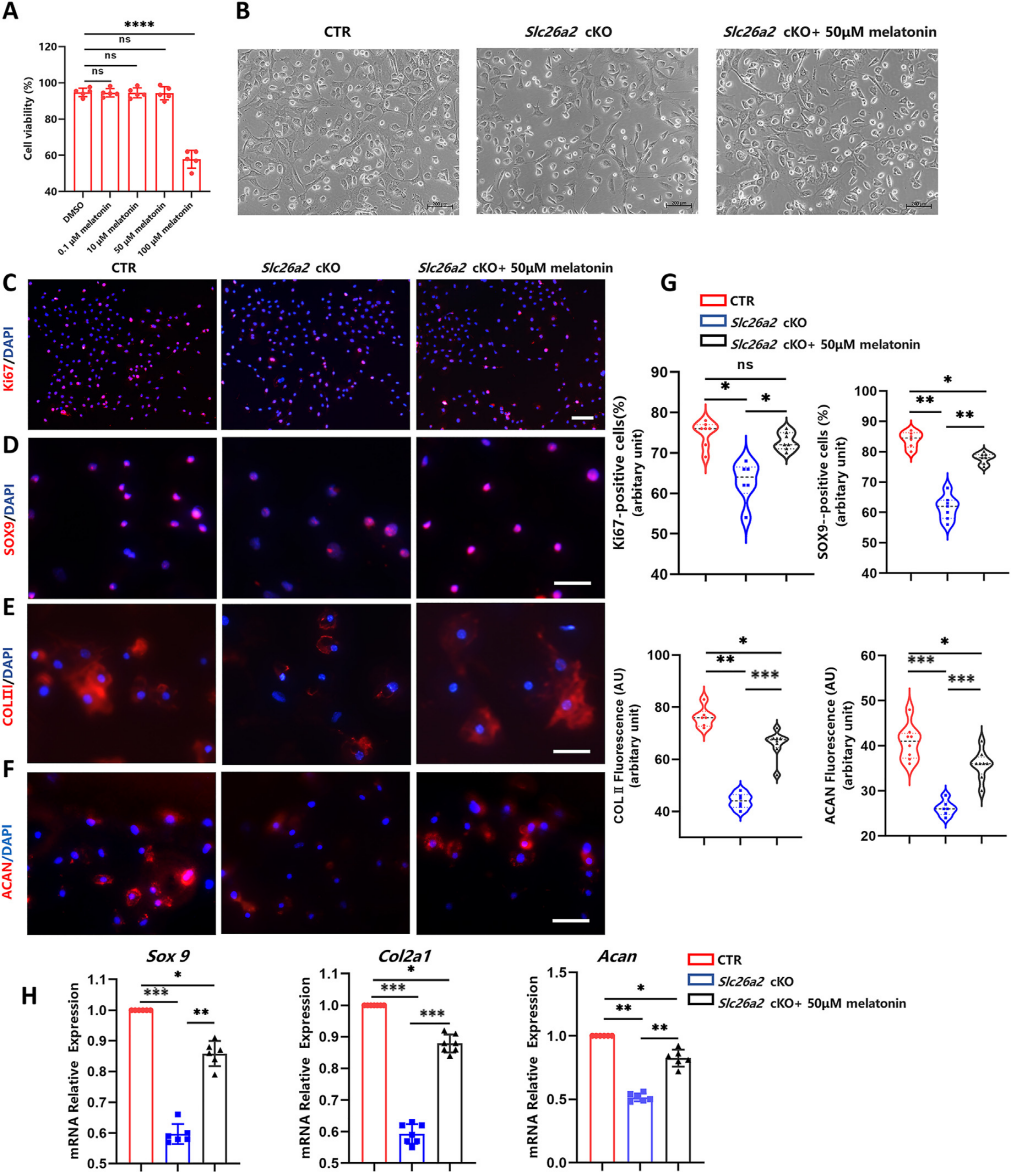
Melatonin promotes proliferation and expression of chondrocyte marker genes of *Slc26a2*-deficient chondrocytes. **(A)** Primary chondrocyte cells were subjected to treatment with different melatonin concentrations, spanning from 0.1 to 100 μM, for 72 h. CCK8 assay revealed that 0.1, 10, and 50 μM melatonin treatments had no impact on cell viability, and the viability of cells notably decreased with the application of 100 μM melatonin. **(B)** The recorded cell morphology of each group was representative. Scale bars, 200 μm. **(C)** Ki67 immunostaining of chondrocytes. Scale bars, 200 μm. **(D**–**F)** Immunofluorescent assessments were conducted on chondrogenic markers SOX9, COL II, and ACAN. Scale bars, 200 μm. **(G)** Measurement of cells expressing Ki67 and SOX9 and chondrocytes labeled with COL II and ACAN. **(H)** Real-time quantitative PCR was employed to analyze the expression levels of *Sox9*, *Col2a1*, and *Acan* mRNA. Statistical significance was assessed through One-way ANOVA followed by Tukey's multiple comparisons test. The results are presented as mean ± standard deviation, with ∗*P* < 0.05, ^∗∗^*P* < 0.01, ^∗∗∗^*P* < 0.001, and ^∗∗∗∗^*P* < 0.0001 indicating statistical significance, while “ns” denotes no statistical significance. SOX9, SRY-box transcription factor 9; COL II, collagen type II; ACAN, aggrecan; SLC26A2, Solute carrier family 26 member 2; CTR, cre-negative control.

The cytotoxicity of melatonin on chondrocytes was evaluated using the CCK8 assay. Primary mouse rib chondrocytes were exposed to varying concentrations of melatonin (0.1, 10, 50, and 100 μM) for 72 h. As shown in Figure 1A, melatonin at concentrations of 0.1, 10, and 50 μM did not significantly affect cell viability, whereas 100 μM melatonin markedly reduced cell viability. Therefore, 50 μM was selected as the optimal dose for further *in vitro* intervention. After treatment with 50 μM melatonin, the cell number increased, compared with the normal group, and the treated chondrocytes tended to maintain a polygonal shape (Fig. 1B). These findings demonstrated the beneficial effects of melatonin on both cell survival and the preservation of chondrocyte morphology. Ki67 staining exhibited a notable decrease in the number of proliferating *Slc26a2*-deficient chondrocytes. However, after treatment with 50 μM melatonin, the number of labeled proliferating cells notably increased (Fig. 1C). During chondrogenic differentiation, SOX9 plays a crucial role in regulating cartilage development and consequently affects the expression of genes that influence chondrocyte formation.^19^ Immunostaining results indicated a reduction in the number of SOX9-positive cells upon *Slc26a2* deficiency, which was significantly recovered after treatment with 50 μM melatonin. Similarly, the expression levels of chondrocyte anabolic markers, COLII and AGGRECAN (ACAN), were significantly diminished in *Slc26a2*-deficient chondrocytes, whereas a substantial increase was observed after treatment with 50 μM melatonin (Fig. 1D–G). These results were corroborated by quantitative PCR analysis with RNA extraction from mouse growth plates (Fig. 1H). In summary, these results clearly indicate the ameliorative effects of melatonin on impaired anabolism and proliferation of *Slc26a2*-deficient chondrocytes.

### Melatonin attenuates activation of UPR induced by *Slc26a2* deficiency

**Figure 2:**
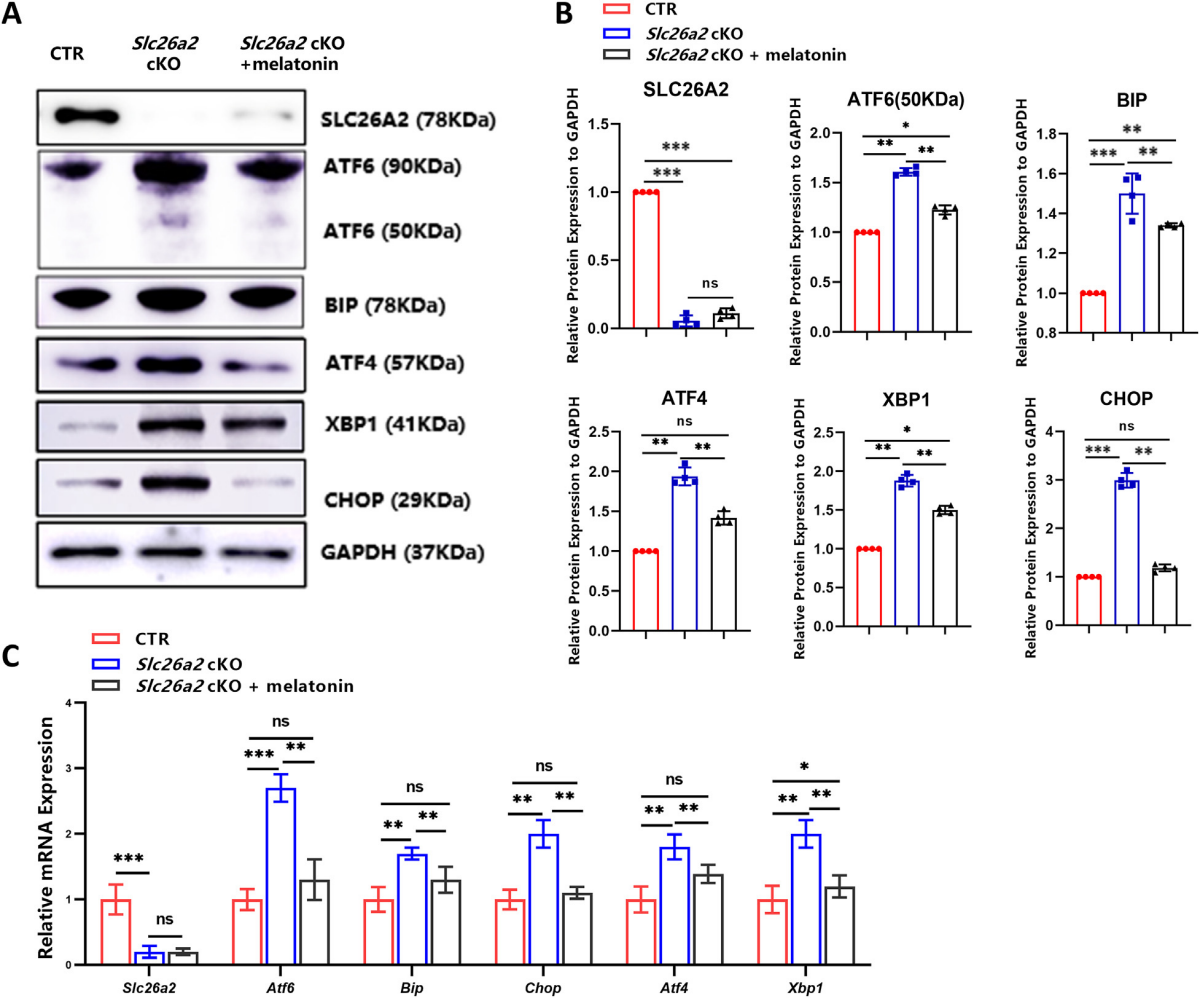
Melatonin attenuates UPR activation in *Slc26a2-*deficient chondrocytes. **(A)** Western blotting revealed that melatonin inhibited the excessive expression of UPR markers in *Slc26a2-*deficient chondrocytes. **(B)** Statistical analyses of western blot results using Image J software. **(C)** Real-time quantitative PCR analysis of UPR markers at mRNA levels in *Slc26a2*-deficient chondrocytes. Statistical significance was assessed through One-way ANOVA followed by Tukey's multiple comparisons test. The results are presented as mean ± standard deviation, with ∗*P* < 0.05, ^∗∗^*P* < 0.01, ^∗∗∗^*P* < 0.001, and ^∗∗∗∗^*P* < 0.0001 indicating statistical significance, while “ns” denotes no statistical significance. UPR, unfolded protein response; CTR, cre-negative control; SLC26A2, Solute carrier family 26 member 2; ATF4/6, transcription factor 4/6; XBP1, X-box binding protein 1; CHOP, C/EBP homologous protein.

We previously reported activation of UPR upon SLC26A2 deficiency.^2^ To quantitatively assess UPR in *Slc26a2*-deficient chondrocytes cultured *in vitro*, we examined the expression levels of key UPR markers in primary chondrocytes and observed an overall up-regulation of ATF6, XBP1, ATF4, BiP, and CHOP at protein levels (Fig. 2A, B). Notably, expression of these markers were all significantly down-regulated following melatonin treatment (Fig. 2A, B). These findings were further confirmed by quantitative PCR analysis of RNA extracted from the chondrocytes (Fig. 2C). Collectively, these results highlighted the substantial impact of melatonin in attenuating activation of UPR upon *Slc26a2* deficiency in chondrocytes.

### Melatonin suppresses cytoplasmic calcium overload *of Slc26a2-deficient* chondrocytes

**Figure 3:**
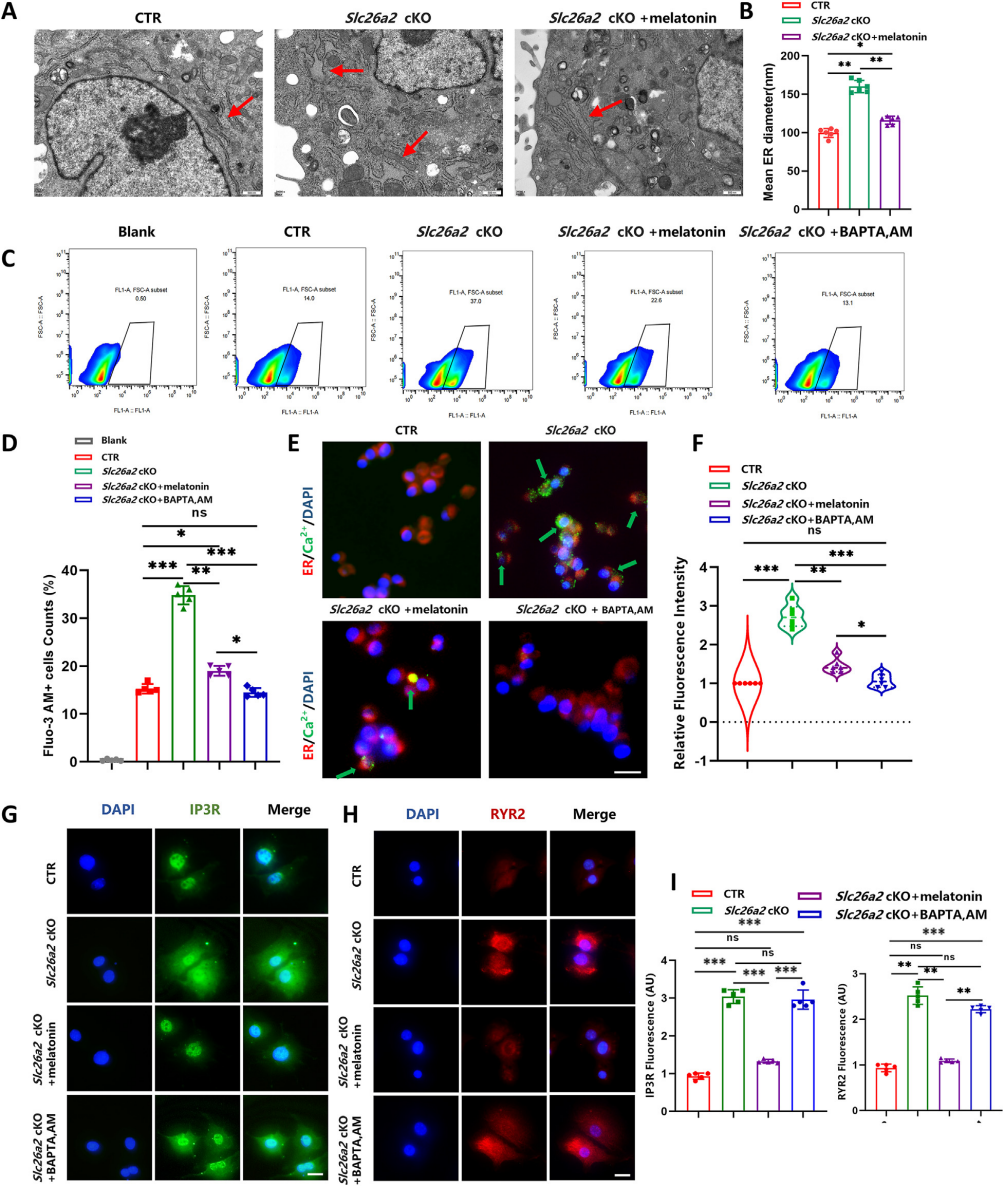
Melatonin inhibits Ca^2+^ overload induced by ER stress upon *Slc26a2* deficiency. **(A)** Representative transmission electron microscopy images of ER. **(B)** Statistical analysis of endoplasmic reticulum diameter. **(C, D)** Flow cytometry analysis of intracellular calcium concentration. **(E, F)** Co-staining of Fluo-3 AM (green), a fluorescent Ca^2+^ indicator, with ER tracker (red) and statistical analysis. Scale bar: 100 μm. **(G–I**) Immunostaining of ER luminal calcium release channel proteins, IP3R and RYR2, and statistical analysis. Scale bar: 100 μm. Statistical significance was assessed through One-way ANOVA followed by Tukey's multiple comparisons test. The results are presented as mean ± standard deviation, with ∗*P* < 0.05, ^∗∗^*P* < 0.01, ^∗∗∗^*P* < 0.001, and ^∗∗∗∗^*P* < 0.0001 indicating statistical significance, while “ns” denotes no statistical significance. ER, endoplasmic reticulum; SLC26A2, Solute carrier family 26 member 2; CTR, cre-negative control; IP3R, inositol 1,4,5-trisphosphate receptor; RYR2, ryanodine receptor 2.

ER stress in chondrocytes was reported to induce calcium overload, ultimately leading to cell death.^20^ Maintaining an appropriate calcium balance is crucial for preserving the functionality of chondrocytes. Transmission electron microscopy imaging was employed for a comprehensive examination of alterations in intracellular structures, which revealed an extended ER within *Slc26a2*-deficient chondrocytes. ER morphology was partially restored after melatonin treatment (Fig. 3A, B). To confirm whether calcium overload was induced, we employed Fluo-3 AM, a fluorescent Ca2^+^ indicator, to assess intracellular calcium concentration, and BAPTA-AM, a cell-permeable Ca^2+^ chelator, to establish a negative control by chelating intracellular Ca^2+^.^21^ Flow cytometry assays revealed pronounced Ca^2+^ overload in *Slc26a2*-deficient chondrocytes with a significant increase in Fluo-3 AM-positive cells, which was partially reversed by treatment with melatonin and pretreatment with BAPTA-AM (Fig. 3C and D). To visualize alterations in cytoplasmic calcium concentration, we co-stained the ER-Tracker Red and Fluo-3 AM (Green). Following melatonin treatment, there was a partial restoration of the significantly elevated free calcium content observed in the cytoplasm of *Slc26a2*-deficient chondrocytes (Fig. 3E, F). Furthermore, the ER luminal Ca^2+^ channel proteins, IP3R (inositol 1,4,5-trisphosphate receptor) and RYR2 (ryanodine receptor 2) were significantly up-regulated in *Slc26a2-*deficient chondrocytes, which was also partially reversed after melatonin treatment (Fig. 3G–I). These experiments confirmed that melatonin inhibits Ca^2+^ overload induced by ER stress upon *Slc26a2* deficiency.

### Melatonin inhibits cell death in *Slc26a2*-deficient chondrocytes

**Figure 4:**
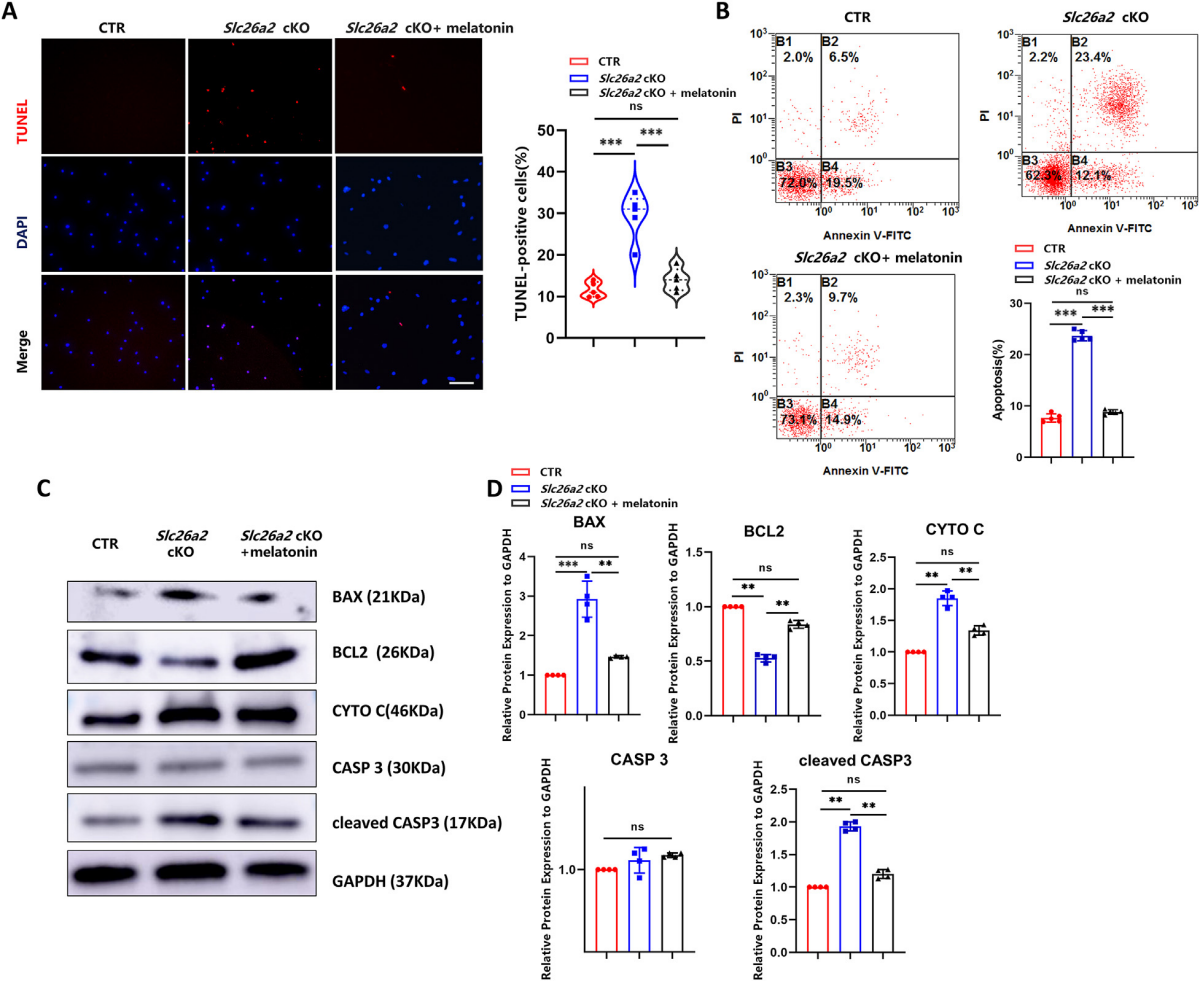
Melatonin attenuates apoptosis of *Slc26a2*-deficient chondrocytes. **(A)** Representative images and statistical analysis of TUNEL cell death assay. Scale bar: 50 μm. **(B)** Flow cytometry analysis of chondrocyte apoptosis. **(C)** Western blotting analysis of pro- and anti-apoptotic proteins. **(D)** Statistical analysis of western blotting data using the Image J software. Statistical significance was assessed through One-way ANOVA followed by Tukey's multiple comparisons test. The results are presented as mean ± standard deviation, with ∗*P* < 0.05, ^∗∗^*P* < 0.01, ^∗∗∗^*P* < 0.001, and ^∗∗∗∗^*P* < 0.0001 indicating statistical significance, while “ns” denotes no statistical significance. SLC26A2, Solute carrier family 26 member 2; CTR, cre-negative control; BAX, Bcl-2 associated X-protein; CYTO C, cytochrome c; CASP3, caspase 3; BCL2, B-cell lymphoma-2.

Given that ER stress and Ca^2+^ overload could lead to programmed cell death, we detected chondrocyte apoptosis by TUNEL assay. Compared with untreated *Slc26a2*-deficient chondrocytes, 50 μM melatonin treatment significantly inhibited cell death caused by *Slc26a2* deficiency (Fig. 4A). In concert with this, flow cytometry also revealed that the apoptotic rate of *Slc26a2-*deficient chondrocytes was 23.4%, which decreased to 9.7% after melatonin treatment (Fig. 4B). Moreover, western blot results indicated an elevated expression of the pro-apoptotic proteins, BAX (Bcl-2 associated X-protein), cytochrome c, and cleaved CASP3 (caspase 3), along with inhibited expression of the anti-apoptotic protein BCL2 (B-cell lymphoma-2) in *Slc26a2*-deficient chondrocytes, compared with those of controls. Following melatonin treatment, the mitigating effects on cell death upon *Slc26a2* deficiency were elicited, as evidenced by down-regulated pro-apoptotic proteins and up-regulated anti-apoptotic proteins (Fig. 4C, D). These data suggested that melatonin could significantly inhibit cell death in *Slc26a2*-deficient chondrocytes.

### Melatonin ameliorates skeletal dysplasia of cartilage-specific *Slc26a2* knockout mice

**Figure 5:**
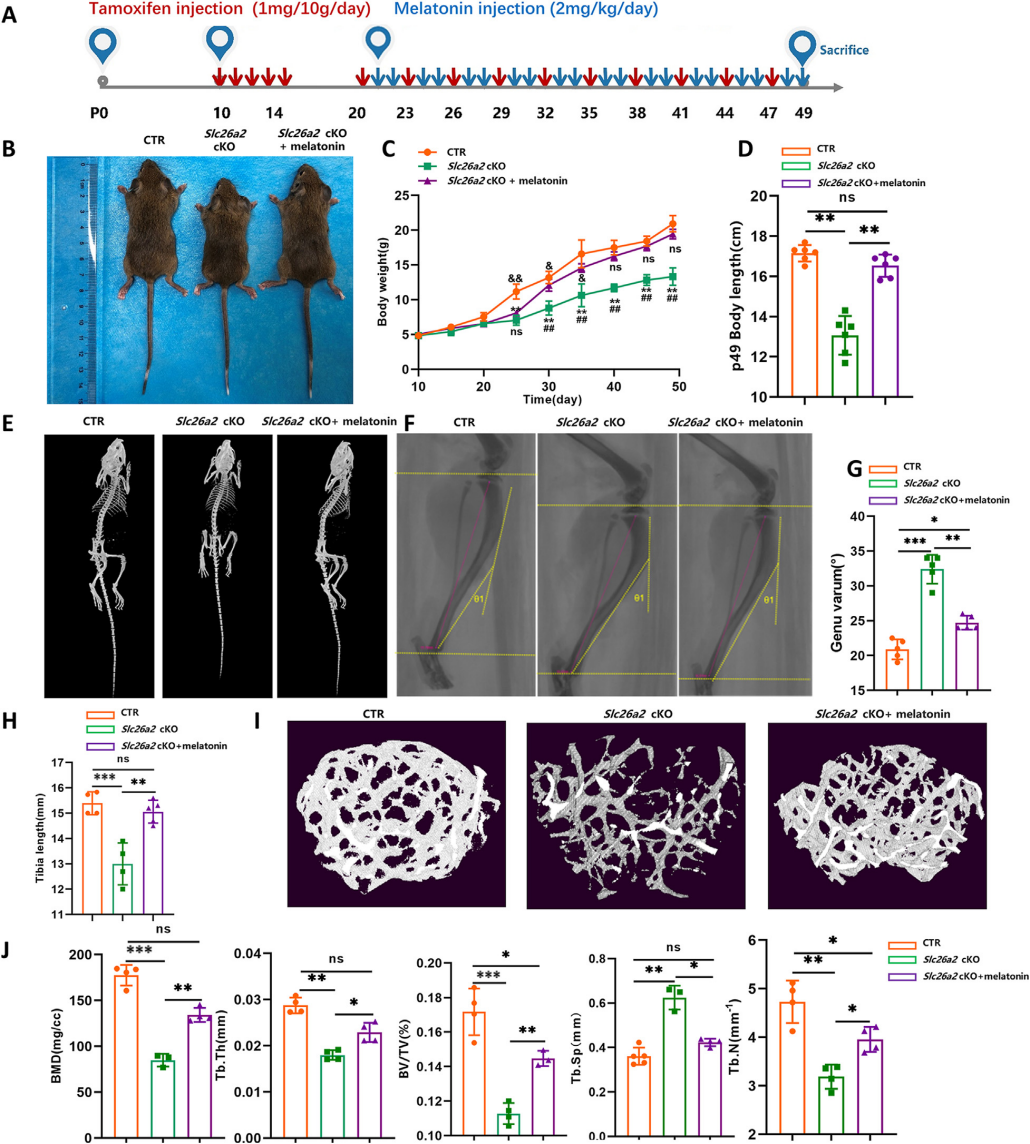
Melatonin ameliorates skeletal dysplasia of *Col2a1-CreER*^*T2*^;*Slc26a2*^*fl/fl*^ mice. **(A)** Overview of the drug delivery plan. Tamoxifen was administered to mice at a dosage of 1 mg per 10 g body weight per day, for consecutive 5 days from P10 to P14 and then every three days from P10 to P49. Melatonin was given from P10 to P49. **(B)** Gross appearance of mice at 7 weeks of age following the administration of tamoxifen and melatonin treatment. **(C, D)** Statistical analyses of body weight and body length. **(E)** Micro-CT reconstruction of the skeleton at P49. **(F–H)** Tibial radiography and statistical analysis. **(I, J)** Micro-CT assessment of bone microstructure and statistical analysis. Statistical significance was assessed through One-way ANOVA followed by Tukey's multiple comparisons test. The results are presented as mean ± standard deviation, with ∗*P* < 0.05, ^∗∗^*P* < 0.01, ^∗∗∗^*P* < 0.001, and ^∗∗∗∗^*P* < 0.0001 indicating statistical significance, while “ns” denotes no statistical significance. SLC26A2, Solute carrier family 26 member 2; CTR, cre-negative control.

Given perinatal lethality of cartilage-specific ablation of *Slc26a2* in mice,^2^ we constructed the *Col2a1-CreER*^*T2*^;*Slc26a2*^*fl/fl*^ mouse line (*Slc26a2* cKO) to induce postnatal *Slc26a2* ablation. Melatonin was administered subcutaneously from P20 to P49 (Fig. 5A, B). In line with *in vitro* mitigating effects, melatonin treatment significantly increased the body weight and body length of *Slc26a2* cKO mice (Fig. 5C, D). Micro-CT analysis revealed that *Slc26a2* cKO mice had significantly shorter limb and spine than control mice (Fig. 5E), and tibial radiography revealed shortened tibia length and tibial genu varum in *Slc26a2* cKO mice (Fig. 5F–H). Melatonin intervention not only resulted in significantly improved limb and spine length but also partially rescued abnormal tibial curvature of *Slc26a2* cKO mice (Fig. 5F–H). Next, we analyzed the distal femur trabeculae using micro-CT. *Slc26a2* cKO mice exhibited a significantly reduced number of femur trabeculae, decreased bone mineral density, bone volume relative to total tissue volume, trabecular number, and trabecular thickness, along with increased trabecular bone separation. Notably, those altered parameters of bone microstructure were significantly ameliorated by melatonin intervention (Fig. 5I, J). Taken together, these findings indicated that melatonin could effectively rescue defective formation and abnormal morphology of bone in *Slc26a2* cKO mice.

### Melatonin attenuates ER stress and cell death in growth plate cartilage of *Slc26a2* cKO mice

**Figure 6:**
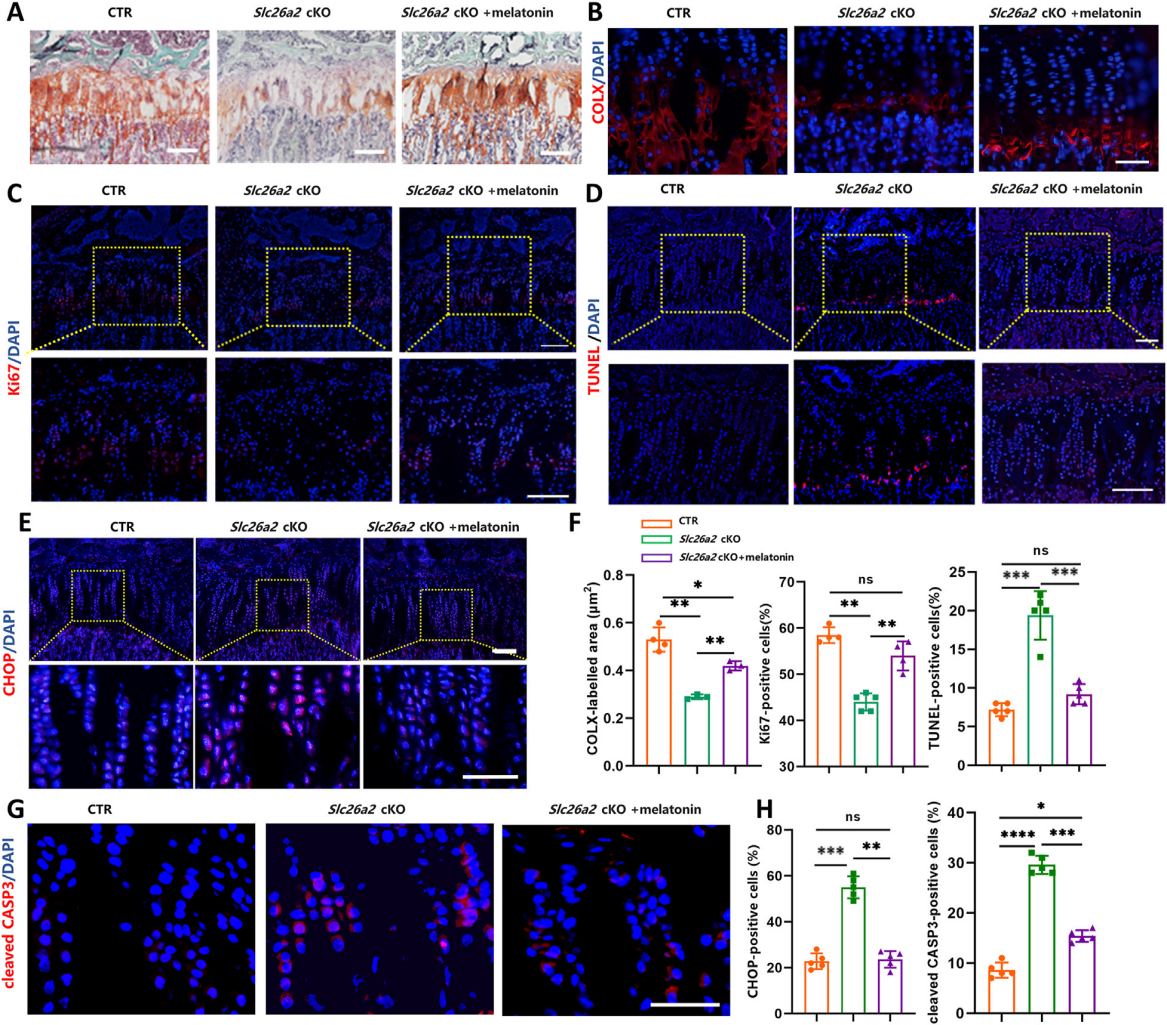
Melatonin elicits a mitigating effect on ER stress and cell death *in vivo* caused by SLC26A2 deficiency. **(A)** Safranin O staining of the tibial growth plate at P49. Scale bar: 100 μm. **(B)** Representative images of COL X immunostaining (red) on the tibial sections. Scale bar: 100 μm. **(C, D)** Representative images of Ki67 immunostaining (red) and TUNEL staining (red) on the tibial sections. Scale bar: 50 μm. **(E)** Representative images of immunostaining of ER stress maker CHOP (red). Scale bar: 50 μm. **(F)** Statistical analyses of cell percentage of Col10a1-, Ki67- and TUNEL-labeled cells in growth plate cartilage. **(G)** Representative images of cleaved CASP3 immunostaining (red). Scale bar: 150 μm. **(H)** Statistical analyses of cell percentage of CHOP- and cleaved CASP3-labeled cells in growth plate cartilage. Statistical significance was assessed through One-way ANOVA followed by Tukey's multiple comparisons test. The results are presented as mean ± standard deviation, with ∗*P* < 0.05, ^∗∗^*P* < 0.01, ^∗∗∗^*P* < 0.001, and ^∗∗∗∗^*P* < 0.0001 indicating statistical significance, while “ns” denotes no statistical significance. ER, endoplasmic reticulum; SLC26A2, Solute carrier family 26 member 2; CTR, cre-negative control; CHOP, C/EBP homologous protein; COL X, collagen X; CASP3, caspase 3.

To assess the mitigating effects of melatonin on ER stress and cell death of chondrocytes, we performed histological analysis with tibial growth plate sections. Safranin O staining indicated a decline in cellularity of both proliferative and hypertrophic zones of growth plate cartilage from *Slc26a2* cKO mice, compared with that of control mice. Remarkably, the administration of melatonin effectively increased the number of chondrocytes within the growth plate (Fig. 6A). Furthermore, the range of COL X expression in the growth plates of *Slc26a2* cKO mice was significantly reduced in comparison to the controls, indicating impaired cell maturation due to *Slc26a2* deficiency. Melatonin treatment elicited a mitigating effect on chondrocyte maturation, as evidenced by a significantly expanded COL X expression range (Fig. 6B). In addition, immunostaining of Ki67 and TUNEL assays showed that the number of Ki67-positive cells within the proliferative zone of *Slc26a2* cKO mice was significantly reduced, whereas an increase in the number of TUNEL-positive cells was observed in the hypertrophic zone of the growth plate. In mice receiving melatonin intervention, Ki67-positive cells significantly increased in the proliferating zone while apoptotic cells decreased in the hypertrophic zone (Fig. 6C, D). CHOP activation during ER stress is crucial for chondrocyte apoptosis.^22^ Hence, we assessed the expression of CHOP and the apoptotic marker cleaved CASP3 in the chondrocytes of the tibial growth plate. Remarkably, melatonin treatment decreased the percentage of chondrocytes positive for CHOP and cleaved CASP3 in *Slc26a2* cKO mice (Fig. 6E–H), indicative of a mitigating effect on ER stress and cell death caused by *SLC26A2* deficiency.

## Discussion

Congenital skeletal dysplasia exhibits diverse phenotypes arising from genetic alterations that disrupt the development and homeostasis of the skeleton, leading to various anomalies in the form and dimensions of individual skeletal components.^23^ Researchers have extensively investigated the pathogenic mechanisms using genetically modified mouse lines, yielding valuable insights for drug development. In particular, the FGFR3 inhibitor has progressed to phase III THOR trial, highlighting its potential for clinical applications.^24^, ^25^, ^26^ Our previous study revealed that *SLC26A2* deficiency triggered activation of the ATF6 pathway, ultimately resulting in overactivation of the FGFR3 signaling cascade.^2^ We repurposed the FGFR3 inhibitor NVP-BGJ398 to treat SLC26A2-deficient chondrodysplasias, and the treatment only partially ameliorated the phenotype. This finding suggests that there could be other complex factors or signaling pathways involved, in addition to the FGFR3 signaling pathway. As specialized secretory cells, chondrocytes are particularly susceptible to ER stress owing to the synthesis and secretion of extracellular matrix proteins by the ER.^27^^,^^28^ In this study, we discovered that ER stress not only overactivated FGFR3 but also caused cytoplasmic calcium overload, jointly leading to increased cellular apoptosis.

Melatonin, renowned for its anti-oxidant and anti-inflammatory attributes, has attracted attention for its therapeutic potential.^29^^,^^30^ Recently, there has been growing interest in the regulatory role of melatonin in ER stress.^31^, ^32^, ^33^ Guan et al highlighted the potential of melatonin in treating non-alcoholic fatty liver disease through the MT2 (metallothionein 2)/cAMP (cyclic adenosine monophosphate)/PKA (protein kinase A)/IRE1 (inositol-requiring enzyme 1) pathway, improving iron homeostasis, inhibiting lipid peroxidation, and mitigating ER stress.^34^ Aouichat et al demonstrated melatonin's anti-obesogenic properties and renal protective effects by suppressing ER stress and the IRE1α/JNK (c-Jun N-terminal kinase) apoptotic pathway in Zücker diabetic fatty rats, potentially offering relief from ER stress-related kidney damage in obesity and diabetes.^35^ Therefore, melatonin is emerging as a promising mitigator of ER stress, demonstrating its potential to alleviate cellular stress and promote overall cellular survival across a spectrum of medical conditions.^36^, ^37^, ^38^ In our study, using *Slc26a2*-deficient chondrocytes induced by 4OH-Tamoxifen and an *in vivo* mouse model mimicking postnatal *SLC26A2*-deficient chondrodysplasia, we observed that melatonin successfully suppressed the expression of ER stress markers upon *Slc26a2* deficiency. The reduced expression of ATF6, XBP1, ATF4, BiP (GRP78), and CHOP indicated an inhibitory effect of melatonin on UPR signaling pathways. The maintenance of an optimal luminal Ca^2+^ concentration in the ER is crucial for the correct folding of secretory proteins, a balance susceptible to disruption by stressors.^20^

During the early stages of ER stress, the activation of the channel proteins IP3R and RYR2 on the ER membrane results in the release of Ca^2+^ into the cytoplasm.^20^ This process facilitated cation transport between organelles,^39^ enhanced oxidative respiratory chain activity, and stimulated mitochondrial ATP production.^40^ Luciani et al revealed that ER Ca^2+^ channels, particularly IP3Rs and ryanodine receptors, regulate beta cell susceptibility to ER stress, highlighting mitochondrial involvement in apoptosis owing to disrupted Ca^2+^ homeostasis.^41^ However, sustained activation of ER stress could hinder cellular metabolism, potentially preventing the restoration of normal ER function and further leading to cell death.^42^ Our findings show that melatonin, by mitigating the detrimental effects of ER stress, reduced intracellular Ca^2+^ overload. Concordantly, melatonin significantly inhibited the expression of pro-apoptotic proteins, including BAX, cleaved CASP3, and CHOP, while enhancing the expression of the anti-apoptotic protein BCL2.

Our finding supported the effectiveness of melatonin in treating *SLC26A2-*associated skeletal disorders, emerging as a valuable addition to the therapeutic approaches. It is noteworthy that melatonin indeed reduced the expression of UPR markers in *Slc26a2*-deficient chondrocytes, which certainly could not exclude the possibility of other proliferation-promoting mechanisms being involved.^43^, ^44^, ^45^ Further studies are necessary to fully elucidate melatonin's multifaceted roles in regulating chondrocyte's function and cell fate. Our study underscored the importance of continued research and development to harness the full therapeutic potential of melatonin for addressing *SLC26A2*-related skeletal conditions.

## Ethics approval

The Animal Research Ethics Committee of the Fourth Military Medical University thoroughly reviewed and approved the animal study.

## Funding

This research was financially supported by the 10.13039/501100001809National Natural Science Foundation of China (No. 82272442, 82372361) and the Key Industrial Chain Program of Shaanxi, China (No. 2022ZDLSF02-12).

## Author contributions

P.L. and Q.J. conceived this research, comprehensively reviewed general references, and meticulously analyzed data. P.L., C.Z., J.Y.H., D.W., W.G.L., X.H., and C.X.Z. discussed the data. Y.L. and Z.J.L. provided revisions to the manuscript.

## Data availability

The authors commit to providing raw data to support the conclusions of this article without reservation.

## Conflict of interests

No potential conflict of interests was disclosed.
